# Understanding and controlling filamentous growth of fungal cell factories: novel tools and opportunities for targeted morphology engineering

**DOI:** 10.1186/s40694-021-00115-6

**Published:** 2021-08-23

**Authors:** Vera Meyer, Timothy Cairns, Lars Barthel, Rudibert King, Philipp Kunz, Stefan Schmideder, Henri Müller, Heiko Briesen, Anna Dinius, Rainer Krull

**Affiliations:** 1 Chair of Applied and Molecular Microbiology, Institute of Biotechnology, Technische Universität Berlin, Straße des 17. Juni 135, 10623 Berlin, Germany; 2 Chair of Measurement and Control, Institute of Chemical and Process Engineering, Technische Universität Berlin, Straße des 17. Juni 135, 10623 Berlin, Germany; 3 Chair of Process Systems Engineering, School of Life Sciences, Technical University of Munich, Gregor- Mendel-Str. 4, 85354 Freising, Germany; 4 Institute of Biochemical Engineering, Technische Universität Braunschweig, Rebenring 56, 38106 Brunswick, Germany; 5 Center of Pharmaceutical Engineering, Technische Universität Braunschweig, Franz-Liszt-Str. 35a, 38106 Brunswick, Germany

**Keywords:** Filamentous fungi, *Aspergillus niger*, Polar growth, Micromorphology, Macromorphology, X-ray micro-computed tomography, Pellet, Co-expression network, Hypothetical proteins, Rheology, Modelling

## Abstract

Filamentous fungal cell factories are efficient producers of platform chemicals, proteins, enzymes and natural products. Stirred-tank bioreactors up to a scale of several hundred m³ are commonly used for their cultivation. Fungal hyphae self-assemble into various cellular macromorphologies ranging from dispersed mycelia, loose clumps, to compact pellets. Development of these macromorphologies is so far unpredictable but strongly impacts productivities of fungal bioprocesses. Depending on the strain and the desired product, the morphological forms vary, but no strain- or product-related correlations currently exist to improve process understanding of fungal production systems. However, novel genomic, genetic, metabolic, imaging and modelling tools have recently been established that will provide fundamental new insights into filamentous fungal growth and how it is balanced with product formation. In this primer, these tools will be highlighted and their revolutionary impact on rational morphology engineering and bioprocess control will be discussed.

## Introduction

**Fig. 1:**
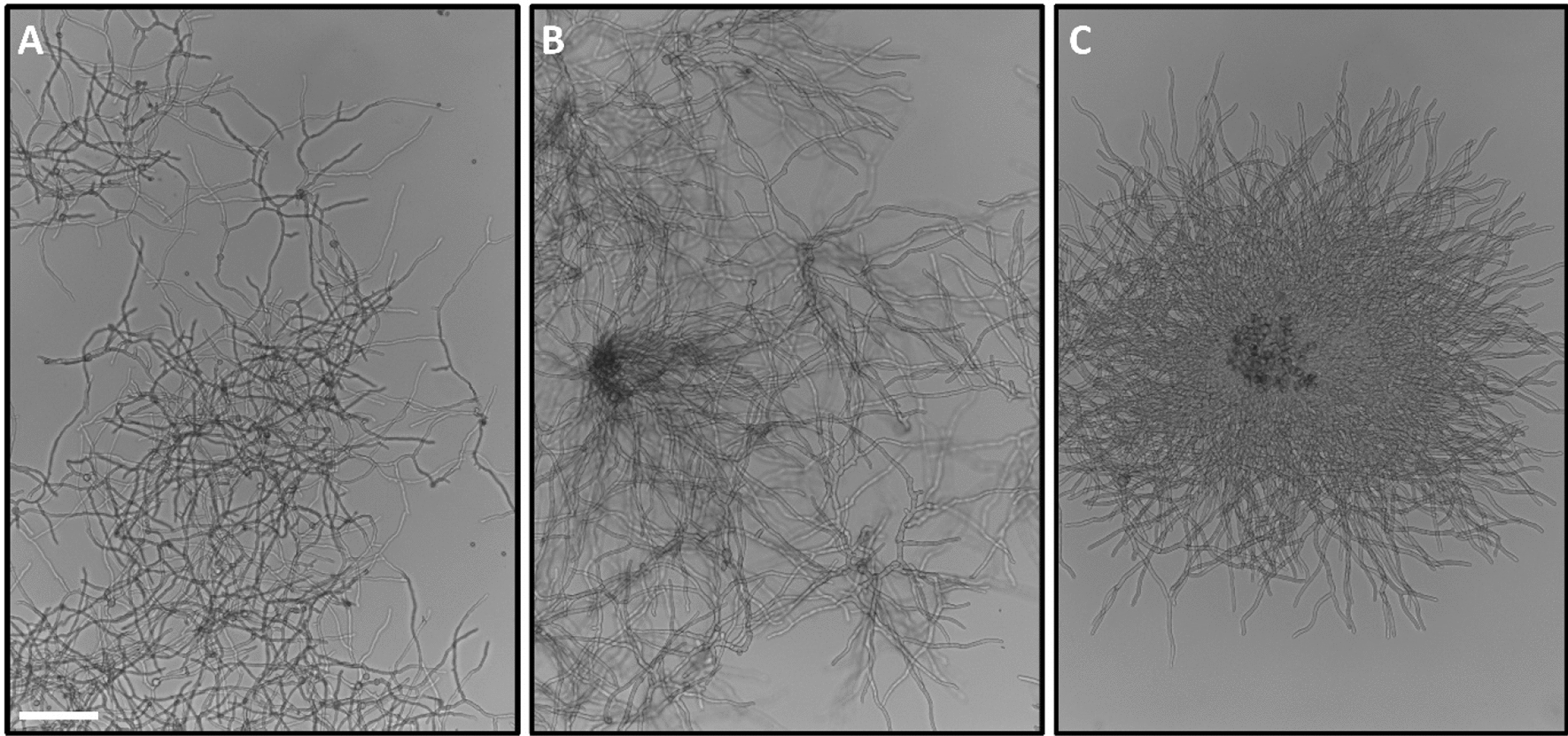
Exemplary microscopic images of *Aspergillus niger*, showing different macromorphologies: **A** dispersed mycelium, **B** clumps, **C **pellets. The scale bar equals 100 µm

**Fig. 2:**
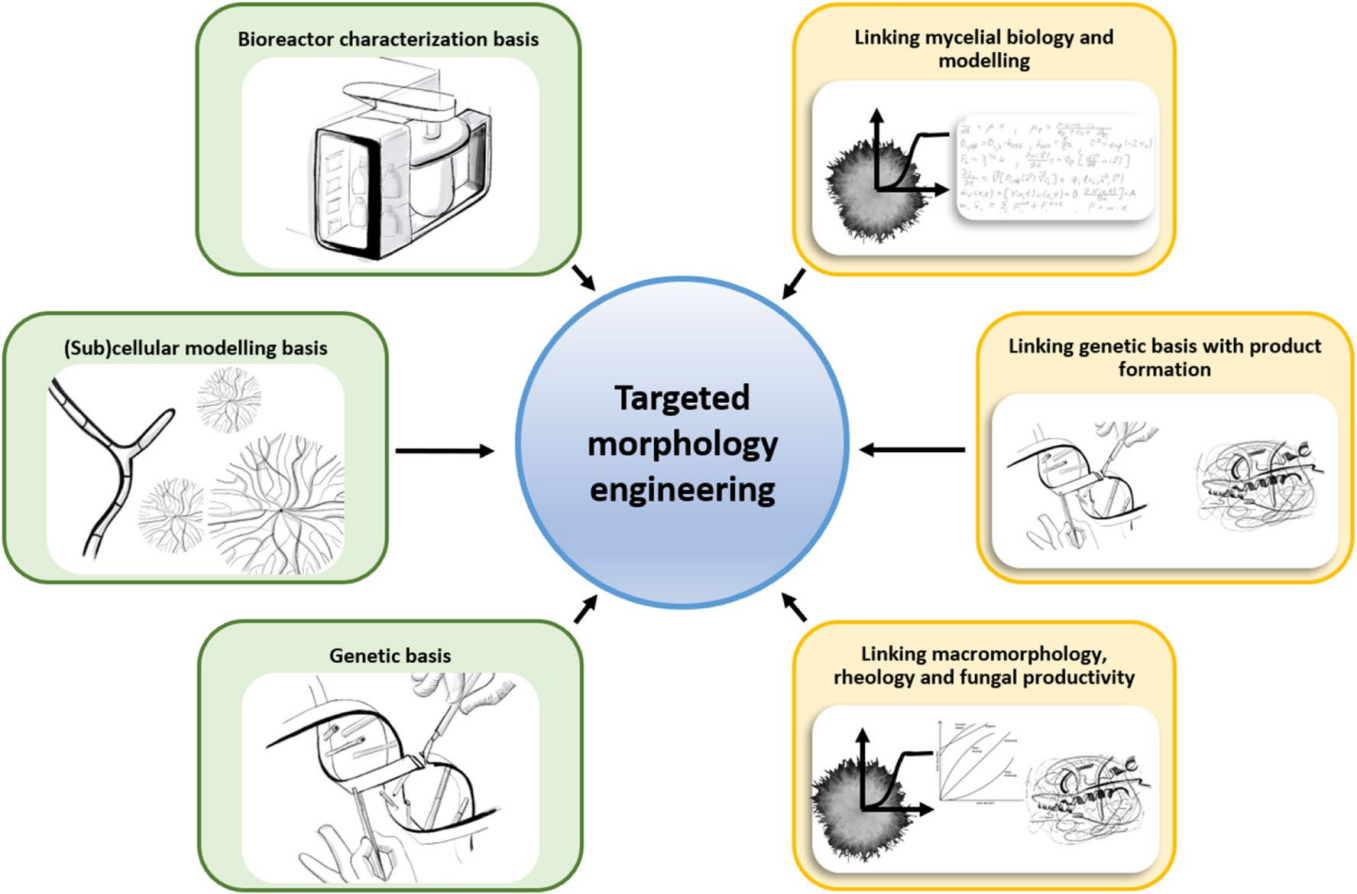
Interconnected concepts and subdisciplines for holistic targeted morphology engineering. Latest advances marked green, upcoming developments marked yellow. Each individual research topic contributes to continued fundamental and applied advances for morphology engineering of filamentous fungi. However, maximal progress can be made when subdisciplines, (e.g. genetics, rheology, subcellular/bioreactor modelling, and mycelial biology) are used to drive new hypotheses and mechanistic discoveries/validation in related disciplines

Fungal biotechnology is a key driver of innovation for the circular economy and contributes to 10 out of the 17 United Nations sustainability goals [1, 2]. Filamentous fungi are masters of both decomposition and biosynthesis, with metabolic versatilities that are unsurpassed in nature. They feed on plant residues from agriculture and forestry, and produce a diverse palette of products which are harnessed by the food, beverage, pharmaceutical, biofuel, textile, feed, automotive, packaging and chemical industries. Usually, filamentous fungi are cultivated under submerged conditions in bioreactors, where they adopt different macromorphologies—dispersed mycelia, clumps or pellets (Fig. 1). The formation of these mycelial architectures depends on the hydrophobicity, vitality, and titre of the spore inoculum. Additionally, cultivation conditions—often summarised under the holistic term *environome*—play a vital role in macromorphology formation, including medium composition, bioreactor geometry, the absence or presence of baffles in shake flasks, hydrodynamic stress due to stirring and aeration, oxygen availability and broth rheology [3, 4]. Thus, the development of macroscopic morphologies is a multifactorial process. In the following, we review both research which has recently progressed and will contribute to our understanding of fungal morphology in the context of product synthesis, and areas which need to be further developed (Fig. 2).

## Why studying the development of dynamic fungal morphologies?

Importantly, the formation of mycelial macromorphologies affects and limits productivities of submerged fungal fermentation processes but are currently unpredictable and thus uncontrollable. The advantage of pellets is that they cause a low viscosity of the cultivation broth and are resistant to shear stress in comparison to the mycelium structure; however, pellets may have dense and inactive cores due to poor oxygen diffusion, which may lead to cell lysis and a loss of the interior pellet structure [5]. Overall, this leads to reduced growth rates and thus reduced growth-associated product formation [6–12]. In contrast, dispersed macromorphologies have other indirect effects on productivity. They grow rapidly and are less limited regarding nutrient transport but are more susceptible to shear stress. They furthermore provoke a higher medium viscosity, which in turn lowers the oxygen transfer rate [10, 13]. Mycelial clumps are less studied and understood but thought to result from the agglomeration of different hyphal elements, which can further agglomerate to pellets [12]. The vision of fungal biotechnologists is to gain mechanistic insights into the development of these macromorphological structures from the genetic, metabolic, subcellular, hyphal, and process level to rationally design filamentous architectures to maximize productivities of fungal bioprocesses. Therefore, various strategies to generate tailor-made fungal cell morphologies have been pursued in the last decade. They include genetic approaches as well as micro-, macro-particle and salt-enhanced cultivation techniques and are summarised under the term *morphology engineering* [3, 14].

## Three advances of research in the last decade

### Dissecting the genetic basis of filamentous growth

Advances in the genetic toolkit for industrially exploited filamentous fungi, including genome editing [15, 16], improved genetic targeting efficiencies [17] and introduction of tight and tuneable monocistronic and polycistronic Tet-on and Tet-off gene switches [18–20] now make generation of mutant strains in a high-throughput manner an exciting possibility. As such, morphology engineering on the genetic level can be considered routine, given that candidate genes whose function is linked to filamentous growth, branching and shear stress resistance are known. Nevertheless, most fungal genes in a genome lack functional characterization (usually filamentous fungal genomes consist of ≥ 10,000 genes). Near genome level predictions of gene function have become possible via newly developed co-expression network approaches. Exemplarily, novel putative functions were assigned for over 9600 out of the 14,000 predicted proteins in the cell factory *Aspergillus niger* [21]. Furthermore, growth and development of engineered strains in shake flasks or bioreactors can now be quantified by user-friendly, high-throughput analysis tools. For example, stereomicroscopic images of filamentous micro- and macromorphologies are quantified by the ImageJ plugin Morphology of Pelleted and Dispersed growth (MPD), which quantifies the ratio of pelleted:dispersed growth forms, Euclidean pellet parameters, and calculates culture-wide non-dimensional morphology metrics that can be used to correlate fungal morphologies with productivity [22, 23].

### Modelling the (sub)cellular basis of filamentous growth

Despite elevated quality and quantity of gene functional predictions, high-throughput experiments are still indispensable to verify the resulting micro- and macromorphologies as the interplay of all gene products is only partially understood. The occurring processes are not only highly dynamic, but also space-dependent leading to specific morphologies. Thus, the objectives of mathematical modelling efforts would be to unravel the consequences of space-dependency and to predict the 3-dimensional properties of a mycelial architecture depending on the strain, its modification, and the environment. The challenge can be addressed on several scales. For example, subcellular modelling of individual hypha or small mycelia allows the investigation of strain-dependent growth, its relation to the spatial organisation of organelles and branching rates on a very fundamental level [8, 24]. Information obtained by confocal laser scanning microscopy (CLSM) can now be used to fit such models to experimental data. An example would be the description of the secretory vesicle flow in a hypha and a quantitative prediction of the spatio-temporal vesicle accumulation at the tip of *A. niger* [25]. For the description of the morphological development of small mycelia, powerful lattice-free [26] or lattice-based methods [27] are available today, both of which most often do make direct use of biological details but focus only on more fundamental (transport) processes and tip extension and branching. For obtaining calibration data, methods of (time lapse) image analysis are routinely applied [22], and are even coupled to high-throughput cultivation systems [28]. On the level of larger mycelia and pellets, growth and branching rates, and thus, structural properties, are additionally affected by process conditions such as substrate availability and broth rheology. Therefore, new experimental techniques are necessary to assess those modelling scales.

Recently, an exciting tool to structurally characterize fungal macromorphologies became available. X-ray micro-computed tomography (µCT) can be employed for the non-destructive 3-dimensional reconstruction of fungal pellets [29]. Morphometrics then allows the quantification of hyphal lengths, average hyphal diameter, hyphal growth units, porosity, as well as the numbers of tips and branches as shown for the cell factories *A. niger* and *Penicillium chrysogenum*. As discussed above, such structural properties are highly intertwined with the transport properties of oxygen, substrates and products. The structural information obtained from the µCT-analysis forms the basis of detailed diffusion simulations through such networks. While detailed transport simulations through the whole pellet are not currently possible, pore-scale diffusion simulations of selectively sampled regions of the pellet are feasible. Consequently, an effective diffusion coefficient can be calculated and correlated with the morphological properties of pellets [30]. By extending the set of investigated pellets, it was furthermore shown that a generalised correlation can be constructed which is basically dependent on the porosity of the mycelial structure. In addition to the µCT assessment of real pellets from five industrial strains, it became also possible to compute such structures via Monte-Carlo growth simulations from scratch. The large set of computer-generated mycelial structures (> 3000) confirmed the generalised correlation of the effective diffusion coefficient with the porosity [31].

### Characterizing the bioreactor environment

In stirred tank reactors, the dissipation of energy impacts cell growth and metabolism through shearing forces and thus cellular morphology, gas/liquid mass transfer and energy transfer. It also causes fragmentation of pellets and mycelial clumps at high power input. For a long time, the stirrer tip speed concept was used to quantitatively describe fluid mechanical stress. Meanwhile, it was proven that the ratio of the maximum local volumetric power input P/V to the averaged P/V should be used. Furthermore, the energy dissipation/circulation function (EDCF) concept was developed [32, 33] to describe fragmentation processes by the interaction of mycelial aggregates and Kolmogorov eddies. EDCF depends on the energy dissipated in the shear-intensive stirrer zone and the circulation frequency of the passage of mycelial aggregates through this zone. Investigations showed that maximal shear stress, occurring at the vicinity of stirrer tips, is the main factor affecting pellet fragmentation and depends on the stirrer types [34–36]. Importantly, fragmentation occurs when eddies are smaller or equal to the size of pellets. Eddies are the result of turbulent flow which is induced by the stirrer and disintegrate into smaller eddies in a vortex cascade and dissipate as heat [37]. While large eddies carry the pellets in a convective motion, small eddies cause shear gradients. The size of terminal eddies can be calculated according to the Kolmogorov micro-scale, which depends on a power law rate approach with the kinematic viscosity and the mass-related power input [38]. To reduce the shear stress, new stirrer types [36] or wave mixed bioreactors [10] can be applied.

Importantly, filamentous cultivation broths generally show non-Newtonian rheological characteristics, i.e. shear thinning and thus pseudoplastic behaviour [39–41]. Consequently, lowering viscosity with increasing shear rate, P/V and mixing time will decrease, whereas mass and heat transfer will increase [42]. It became state-of-the art to calculate the apparent viscosity in bioreactors; whereby the Metzner and Otto concept [43] is commonly used to calculate laminar flow, and the power concept to calculate transitional and turbulent flow regimes. The latter showed that P/V and not the stirring rate correctly characterises gas/liquid mass transfer and heat transfer in stirred tank reactors [44].

## Three areas ripe for development

### Systems understanding of the genetic basis of filamentous growth and connecting this knowledge with product formation

Despite the recent advances in the genetic and genomic toolkit, a significant and persistent challenge to understanding filamentous fungi as integrated systems is that approximately 50% genes are currently annotated as encoding a ‘hypothetical protein’ [45]. This can significantly limit interpretation of ‘omics datasets. Genome-wide knock-out and gain-of-function libraries consisting of thousands of mutant strains could overcome this limitation in the near future. With decreased DNA synthesis costs, construction of such genome-wide libraries for industrial filamentous fungi is in general feasible; however, this can only be achieved in a community-wide effort as the generation and systematic analysis of such gene libraries are cost, reagent and time intensive. Linking these data with community-driven metabolic network reconstructions for industrial filamentous fungi [46, 47] will, however, considerably improve our understanding of filamentous growth and the metabolic power hidden in fungal genomes. Noteworthy are the most recent insights into the unexpected link between filamentous growth with organic acid and protein production. Genes predicted to function in vesicle transport from the Golgi organelle to the hyphal tip of *A. niger* are surprisingly co-expressed with numerous citric acid cycle genes [48]. Indeed, Tet-on conditional expression mutants demonstrated that expression of these genes is required for citric acid production, thus linking citric acid and protein production for the first time at a genetic level. We anticipate that further interrogation of these gene and metabolic networks will guide gene functional analysis experiments to develop highly efficient filamentous fungal production strains.

### Strengthening the link between mycelial biology and modelling

Routinely analysing a large number of pellets via µCT is still very time-consuming and costly. Also, the resolution limits of about 1 μm for typical µCT restricts broad application. Synchrotron imaging and advancing sample preparation techniques, however, may push the boundaries of such current limitations. Full three-dimensional information will thus be available for the quantification of targeted genetic modifications on mycelial structures, investigating the interplay of growth behaviour with the transport of oxygen, substrates and products within pellets.

With respect to modelling, a seamless multiscale integration of various approaches will become possible. Subcellular models form the basis of branching rates and branching directions to be used in Monte-Carlo simulations of pellet formation [31, 49, 50]. Such single pellet information needs to be transferred to a bioreactor scale, where the heterogeneity of different pellets with respect to pellet diameter, aspect ratio and surface solidity need to be accounted for. Such heterogeneity can be represented in terms of a population balance approach, which already has found use in a more descriptive fashion [51, 52]. Finally, a coupling of such population balance models to computational fluid dynamics models will provide the full picture of an industrially relevant process.

The foundation of this modelling pipeline rests on subcellular models. However, up to now, mathematical models only replicate what is seen in an experiment given a specific strain and focussing on selected fundamental processes. It is still out of reach to predict the quantitative, time-dependent morphological outcomes resulting from genetic modification. To bridge this gap, more refined and detailed models must be built based on the continuously growing biological knowledge, e.g., with respect to polarity establishment at a branching site or cytoskeletal reorganization within fungal hyphae. This knowledge has to be combined with space- and time-resolved data, e.g., from growth chambers connected to a CLSM, to formulate and calibrate more detailed models. Concomitantly, mathematical modelling can help to formulate new biological hypotheses from a different perspective and thus providing new impulses. Today, branching is mostly described by a branching rate. However, when branching had to be modelled on a subcellular level to relate it to a genetic modification, new questions arise. What happens quantitatively to the flow of secretory vessels (SV)? When and how is SV-production upregulated in a (sub)apical or lateral section where the branch is formed? Where and when are septa formed, and how do they relate to branch formation? How do branching and secretory mechanisms differ between phyla? If a refined mathematical description of branching becomes possible with this information, how can it be condensed to a branching frequency for the next level of model abstraction? On that scale, does enhanced shear stress change the distribution of vesicles used in the model to describe length growth and those exploited for product secretion and how can a modified shear stress be produced and studied on a microscopic scale for a single hypha? Hence, many new questions can be formulated and potentially answered through a tight collaboration between fungal biologists and modellers.

### Linking macromorphology, rheology and fungal productivity

Due to the high number of genes to be analysed and the heterogeneous character of fungal macromorphological populations in a bioreactor, there is an increased need for the development of parallel multibioreactor systems at the millilitre scale that allow for high-throughput and that generate reproducible cultivation data with low variance. The development and commercialization of several online monitoring tools for shaken flasks (Respiration Activity MOnitoring System, RAMOS) and microtiter plates (µRAMOS, BioLector) are suitable techniques that allow deep insight into the metabolic state of microbial cultivations and are suitable as scaling parameters that can be determined at an early stage of process development with filamentous microorganisms [53–55].

In addition, most model approaches for predicting the productivity of pellet cultures are linked to a specific bioreactor system and fixed cultivation conditions. Reaction kinetics are averaged almost without exception over the entire heterogeneous pellet population, even if the individual pellets have different morphologies and thus contribute differently to the overall productivity. For the prediction of productivity, transferable models need to be developed, taking into account the distribution of the pellet population independently of the reactor geometry and the operating conditions. For these purposes, integral observation variables, e.g. the average oxygen, biomass or product concentration, have to be traced back to the pellet level, whose properties change with the mechano-chemical interactions with the surrounding cultivation medium. In order to bridge the scales between the pellets and the bioreactor, turbulent flow models must be linked. Pellet-specific and morphology-dependent product formation rate expressions for these interactions have to be derived, calibrated and validated [14]. Also, rheo-morphological parameter correlations are challenging due to the large variability of mycelial structures in submerged cultures. To quantify the interrelationship between fungal macromorphologies, rheology, and protein productivity, non-dimensional morphology metrics have been successfully applied [22, 48, 56]. Through comparison of morphological and rheological data it was evident that dispersed mycelia of *A. niger* are most productive with respect to protein secretion, but also exhibits the highest culture broth viscosity. Given that protein secretion and viscosity are generally negatively correlated, it seems feasible to estimate product formation from rheological data. In another work, it was confirmed that protein secretion is preferred in *A. niger* with dispersed mycelium, but that the same strain favoured citric acid production when pellets were formed [48]. Hence, future fungal strain engineering efforts should assess productivity from various product perspectives as there is no consensus how to correlate cellular morphology, broth rheology and productivity.

## Conclusions

Despite recent advances, a universal model of fungal growth—ranging from dynamic changes in subcellular structures of fungal hyphae to 3-dimensional mycelial growth—does not yet exist. As with the last decade, experimenters and modellers have to go hand in hand to mutually benefit from each other and to jointly develop a holistic understanding of the optimum macromorphology for a given product and a given strain. This future-to-be-developed portfolio of suitable tools should also be transferable to other filamentous systems, e.g., actinomycetes and plant cell cultures.

## Data Availability

Not applicable.
